# The effect of dietary supplementation of salts of organic acid on production performance of laying hens

**DOI:** 10.14202/vetworld.2016.1478-1484

**Published:** 2016-12-30

**Authors:** Ravinder Dahiya, Raj Singh Berwal, Sajjan Sihag, Chandrashekhar Santosh Patil, Lalit

**Affiliations:** 1 Department of Animal Nutrition, Lala Lajpat Rai University of Veterinary and Animal Sciences, Hisar, Haryana, India; 2 Department of Animal Genetics and Breeding, Lala Lajpat Rai University of Veterinary and Animal Sciences, Hisar, Haryana, India

**Keywords:** calcium-propionate, egg production, feed conversion ratio, feed intake, sodium butyrate and layers

## Abstract

**Aim:**

An experiment was conducted to evaluate the effect of supplementing different levels of salts of organic acid in the laying hen’s diet on their production performance and egg quality parameters during a period of 16-week.

**Materials and Methods:**

A total of 140 white leghorn laying hens at 24 weeks of age were randomly distributed to seven dietary treatment groups, i.e. T_1_ (control), T_2_ (0.5% sodium-butyrate), T_3_ (1.0% sodium-butyrate)_,_ T_4_ (1.5% sodium-butyrate), T_5_ (0.5% calcium-propionate), T_6_ (1.0% calcium-propionate) and T_7_ (1.5% calcium-propionate) consisting of 5 replications of 4 birds each in each treatment and housed in individual cages from 24 to 40 weeks of age. Feed intake, percent hen-day egg production, egg weight, egg mass production, feed conversion ratio (FCR), and economics of supplementation of salts of organic acids in layers’ ration were evaluated.

**Results:**

The dietary supplementation of salts of organic acids did not significantly affect the feed intake (g/day/hen) and body weight gain (g). Different levels of supplementation significantly (p<0.05) improved production performance (percent hen-day egg production and egg mass production) as compared to control group. FCR in terms of feed intake (kg) per dozen eggs was lowest (1.83±0.05) in T_4_ and feed intake (kg) per kg egg mass was lowest (2.87±0.05) in T_5_ as comparison to control (T_1_) group. Salts of organic acids supplementation resulted in significant (p<0.05) improvement in FCR. Egg weight was significantly (p<0.05) increased at 0.5% level of salts of organic acids in the diet. The cumulative mean values of feed cost per dozen egg production were Rs. 44.14, 42.40, 42.85, 43.26, 42.57, 43.29 and 43.56 in treatment groups T_1_, T_2_, T_3_, T_4_, T_5_, T_6_ and T_7_, respectively, and reduction in feed cost per kg egg mass production for Rs. 0.52 and 0.99 in groups T_2_ and T_5_, respectively, in comparison to T_1_ group.

**Conclusions:**

It can be concluded that supplementation of salts of organic acids may improve persistency of lay, egg weight, and FCR. From economical point of view, egg production was more profitable at 0.5% level of sodium butyrate and 0.5% level of calcium propionate which reduced the feed cost per dozen eggs and per kg egg mass production without affecting the egg quality.

## Introduction

The poultry industry is a well-organized and fast growing sector of Indian economy. India is emerging as the world’s 2^nd^ largest poultry market with an annual growth of more than 14%, producing 61 million tones or 3.6% of the global egg production and annual growth rate of egg production is 5-8% [1].

Antibiotics have been widely used in poultry production for decades to improve growth rate and feed conversion efficiency; however, their use as growth promoters in the poultry industry has been intensively controversial because of the development of bacterial resistance and potential consequences on the human health [2]. In response to this apparent threat, the European Commission (EC) decided to phase out, and ultimately ban (January 1^st^, 2006), the marketing and use of antibiotics as growth promoters in feed (EC Regulation No. 1831/2003). Now the poultry sector is continuously searching for new feed additives to improve the feed efficiency with minimum deleterious effects on animal health. Organic acids and their salts are generally regarded as safe and have been approved by most member states of European Union to be used as feed additives in the animal production [3]. Organic acid associated with specific antimicrobial activity are short chain acids (C_1_-C_7_) which are widely distributed in nature as normal constituents of plants or animal tissues and also formed through microbial fermentation of carbohydrates predominantly in the ceca of poultry [4]. The advantage of salts over acids is that they are generally odorless and easier to handle in the feed manufacturing process owing to their solid and less volatile form [5]. Organic acids can serve as a meaningful tool to controlling all enteric nonpathogenic and pathogenic especially acid-intolerant bacteria such as *Escherichia coli*, *Salmonella*, and *Campylobacter* species [6]. Apart from the antimicrobial activity, they lower down the gastric pH which accelerates the conversion of pepsinogen to pepsin, which ultimately improves the absorption and digestibility of proteins, amino acids, minerals and serve as substrates in the intermediary metabolism.

Organic acids have made a great contribution to profitability in poultry production and also provided people with nutritious poultry products [7]. Nollet *et al*. [8] and Mahdavi *et al*. [9] reported that a positive effect of sodium butyrate and calcium propionate on laying performance that is conform the variable effect of the additives may be confounded by variations in gut flora and environmental conditions.

Therefore, this investigation was undertaken to study the effect of dietary supplementation of salts of organic acid on production performance in laying hens.

## Materials and Methods

### Ethical approval

All the experimental procedures have been conducted in accordance with the guidelines laid down by the Institutional Ethics Committee.

### Experiment and data structure

The investigation was conducted at poultry farm, Department of Animal Genetics and Breeding, College of Veterinary sciences, LUVAS, Hisar. For this study, 140 single comb white leghorn laying hens at 24 weeks of age were randomly distributed to seven dietary treatment groups, i.e. T_1_ (control), T_2_ (0.5% sodium-butyrate), T_3_ (1.0% sodium-butyrate)_,_ T_4_ (1.5% sodium-butyrate), T_5_ (0.5% calcium-propionate), T_6_ (1.0% calcium-propionate) and T_7_ (1.5% calcium-propionate), consisting of five replications of four birds each in each treatment. Based on the proximate composition and metabolizable energy of feed ingredients the layer’s control ration having maize grain as energy source was formulated as per BIS [10]. All the diets were analyzed for proximate principles [11] and were randomly divided into four groups. The hens were housed individually in cages. All the diets were prepared to be isocaloric and isonitrogenous. They were reared under identical conditions of environment and management of light, water, disease control, etc. Feed and water were supplied ad lib. The study was undertaken from 24 to 40 weeks of age of layers in the first phase of production cycle. The entire duration of the study was divided into eight periods of 14-day each.

For each replicate group, feed intake was measured on weekly basis, whereas egg production data were recorded daily. Random samples of 5 eggs from each treatment (1 egg/replicate) were collected at weekly intervals for measurement of egg weight and egg mass production. Feed conversion ratio (FCR) was expressed as kilogram of feed consumed per dozen egg produced and per kg of egg mass produced for the 8 periods (24-26, 26-28, 28-30, 30-32, 32-34, 34-36, 36-38 and 38-40 weeks of age) of 2 weeks each and cumulative intake of 1-8 periods (24-40 weeks). Average feed cost (Rs.) per dozen egg production and per kg egg mass production during progressive age (weeks) under different dietary treatments calculated at the end of study.

### Statistical analysis

The statistical analysis of data was performed using SPSS 21.0 version of Microsoft [12]. One-way ANOVA was used for the differences between groups. When the p values were significant (p<0.05), a Duncan’s multiple range test was performed [13]. All the data were expressed as mean±standard errors.

## Results and Discussion

The feed intake (g)/bird/day, percent hen-day egg production, egg weight, egg mass production and FCR for 1-8 periods (24-40 weeks). The ingredient and chemical composition of basal diet fed to laying hens of control group have been given in Table-1. The contents of crude protein, crude fiber, ether extract, nitrogen-free extract, and organic matter of basal diet (T_1_) were 18.04%, 4.34%, 3.61%, 66.21% and 92.20%, respectively. The calculated value of ME was 2697.17 kcal/kg feed.

**Table-1:** The ingredients and chemical composition of control diet.

Ingredients	(kg/100 kg feed)
Ingredient composition	
Maize	50
Soybean meal	13
Groundnut cake	7
DORP	12
Rice polish	5
Fish meal	6
Mineral mixture	3
Salt	1
Shell grit	3
Total	100
Nutrients	(% DM basis)
Chemical composition	
CP	18.10
CF	4.28
EE	3.65
Ash	10.21
OM	89.79
NFE	63.76
ME* (Kcal/kg)	2697.17

Spectromix - 10 g/quintal (Each g contained vitamin A - 82,500 IU, vitamin D3 - 12,000 IU, vitamin B2 - 50 mg, and vitamin K - 10 m) Spectrimix-BE - 10 g/q, (Each g contained vitamin B1 - 80 mg, vitamin B6-16 mg, niacin - 120 mg, vitamin B12 - 80 mg, calcium pantothenate - 80 mg, vitamin E - 160 mg, L-lysine HCl - 10 mg, DL methionine - 10 mg, and calcium - 260 mg); (P < 0.01) **Calculated value [27].

* Calculated kcal/kg. CP = Crude protein, CF = Crude fiber, EE = Ether extract, NFE = Nitrogen-free extract, OM = Organic matter, ME = Metabolizable energy, DM = Dry matter, DORP = De Oiled Rice Polish

### Feed intake

The results of the study revealed that the feed intake (g/hen/day) was not significantly affected by the supplementation of different levels (0.5%, 1.0%, and 1.5%) of salts of organic acids in the diet of layers (Table-2). These results are in agreement with Bonos *et al*. [14] and Kaya *et al*. [15] who reported that supplementation of salts of organic acids had no effect on average feed intake and body weight changes. By contrast, Youssef *et al*. [16] reported that supplementation of salts of organic acids significantly increased live body weight in layers.

**Table-2:** Feed intake (g/hen/day) and FCR under different dietary treatments in laying hens.

Parameters (Week)	Treatment						

	T_1_	T_2_	T_3_	T_4_	T_5_	T_6_	T_7_
Feed intake							
24-26	114.64^a^±1.34	114.96^a^±1.06	118.31^c^±1.45	118.50^c^±1.60	114.82^a^±0.77	117.28^b^±1.55	118.14^c^±1.06
26-28	116.82^ab^±1.55	116.58^ab^ 1.01	118.76^b^±1.42	119.04^b^±1.45	116.08^a^±1.35	118.56^b^±1.14	118.75^b^±1.43
28-30	117.35^a^±1.67	118.00^ab^±1.35	120.11^c^±1.37	120.07^c^±1.38	118.92^b^±1.20	119.77^bc^±1.37	120.08^c^±1.47
30-32	119.03^a^±1.25	120.95^b^±1.27	121.90^bc^±1.53	122.71^c^±1.54	119.84^ab^±1.26	121.92^bc^±1.42	122.52^c^±1.78
32-34	121.42^a^±1.19	121.49^a^±1.26	123.61^bc^±1.77	123.96^c^±1.45	122.04^ab^±1.39	123.00^abc^±1.50	123.73^bc^±1.31
34-36	122.92±1.65	123.38±1.25	123.95±1.37	124.61±1.24	122.98±1.27	123.75±1.22	124.48±1.34
36-38	121.67^ab^±1.33	122.34^b^±1.88	123.01^bc^±1.25	123.69^c^±1.30	120.42^a^±1.44	121.67^ab^±1.38	122.17^b^±1.47
38-40	119.11±1.43	120.42±1.26	121.00±1.78	121.61±1.50	118.87±1.44	120.16±1.38	121.18±1.84
Mean FCR (kg feed consumed per dozen of egg production)	119.11±1.42	119.76±1.29	121.33±1.49	121.77±1.43	119.87±1.26	120.76±1.37	121.38±1.46
24-26	1.99^c^±0.07	1.92^b^±0.07	1.90^a^±0.04	1.89^a^±0.04	1.92^b^±0.09	1.92^b^±0.06	1.90^a^±0.07
26-28	2.00^c^±0.08	1.87^b^±0.06	1.85^a^±0.07	1.84^a^±0.08	1.88^b^±0.08	1.88^b^±0.08	1.86^ab^±0.06
28-30	1.96^c^±0.06	1.84^b^±0.04	1.82^a^±0.04	1.80^a^±0.05	1.85^b^±0.09	1.84^b^±0.04	1.81^a^±0.04
30-32	1.94^c^±0.07	1.82^b^±0.04	1.80^ab^±0.08	1.78^a^±0.07	1.83^b^±0.06	1.83^b^±0.08	1.80^ab^±0.07
32-34	1.91^c^±0.04	1.81^b^±0.08	1.79^ab^±0.08	1.77^a^±0.07	1.81^b^±0.08	1.81^b^±0.07	1.79^ab^±0.05
34-36	1.88^c^±0.05	1.77^b^±0.08	1.76^b^±0.07	1.74^a^±0.07	1.78^b^±0.08	1.78^b^±0.08	1.77^b^±0.05
36-38	2.08^c^±0.05	1.95^b^±0.08	1.93^ab^±0.07	1.91^a^±0.07	1.97^b^±0.09	1.96^b^±0.08	1.94^ab^±0.05
38-40	2.13^c^±0.06	1.98^b^±0.07	1.97^ab^±0.07	1.95^a^±0.07	2.00^b^±0.08	1.99^b^±0.09	1.96^a^±0.05
Mean FCR (kg feed consumed per egg mass production)	1.98^c^±0.06	1.87^b^±0.05	1.85^ab^±0.07	1.83^a^±0.05	1.88^b^±0.08	1.87^b^±0.07	1.85^ab^±0.06
24-26	2.89^ab^±0.07	2.80^a^±0.05	2.98^b^±0.06	2.97^b^±0.04	2.79^a^±0.05	2.90^ab^±0.07	2.93^ab^±0.06
26-28	2.93^b^±0.09	2.83^a^±0.04	2.95^b^±0.05	2.94^b^±0.07	2.80^a^±0.04	2.90^ab^±0.07	2.89^ab^±0.05
28-30	2.94^ab^±0.06	2.86^a^±0.08	3.02^b^±0.09	2.97^ab^±0.06	2.90^a^±0.04	2.94^ab^±0.05	2.94^ab^±0.05
30-32	2.95^b^±0.07	2.90^ab^±0.07	3.00^c^±0.07	2.95^b^±0.09	2.84^a^±0.07	2.96^b^±0.06	2.96^b^±0.07
32-34	3.01^b^±0.04	2.95^ab^±0.06	3.07^c^±0.10	2.91^a^±0.07	2.94^ab^±0.09	2.99^b^±0.07	2.98^b^±0.08
34-36	3.06^c^±0.07	2.89^a^±0.05	2.98^b^±0.07	2.96^b^±0.06	2.90^a^±0.04	2.94^ab^±0.05	2.94^ab^±0.08
36-38	3.03^c^±0.05	2.83^ab^±0.05	2.99^c^±0.09	2.72^a^±0.06	2.82^ab^±0.07	2.91^b^±0.06	2.91^b^±0.07
38-40	2.97^a^±0.04	3.02^a^±0.07	3.19^b^±0.09	3.20^b^±0.07	3.00^a^±0.06	3.14^b^±0.06	3.17^b^±0.08
Mean	2.97^b^±0.06	2.88^a^±0.05	3.02^c^±0.07	2.95^b^±0.06	2.87^a^±0.05	2.96^b^±0.06	2.96^b^±0.06

The mean values in same row with different superscripts differ significantly (p<0.05). FCR=Feed conversion ratio

### Percent hen-day egg production

Percent hen-day egg production values were 74.72%, 76.16%, 77.39%, 79.14%, 75.70%, 76.72% and 77.77% in the treatment groups T_1_, T_2_, T_3_, T_4_, T_5_, T_6_ and T_7_, respectively (Table-3). The results of the study unveiled that there was significant (p<0.05) positive effect on percent hen-day egg production by the supplementation of different levels (0.5%, 1.0%, and 1.5%) of salts of organic acids in the diet of layers. Hen-day egg production was highest (79.14) in T_4_ (1.5% sodium butyrate) as compared to all other treatment groups. Similar trends of egg production were observed during different weeks of age of laying hens. The results are in close resemblance with Nollet *et al*. [8] reported a positive effect of sodium butyrate on laying performance increasing from 83.1% (control) to 83.8%, 84.3%, 84.8% and 86.1% (50, 100, 250 and 500 ppm of sodium butyrate supplementation), respectively. Moreover, Yesilbag and Colpan [17] observed that dietary supplementation of salts of organic acids had accelerated the laying capacity in 24-28 weeks old laying hens and extended the period of egg production in 36-38 weeks old hens than hens of control group.

**Table-3:** Egg production and egg weight in layers fed diet supplemented with different levels of probiotics and prebiotics during progressive weeks of age.

Parameters (Weeks)	Treatment						

	T_1_	T_2_	T_3_	T_4_	T_5_	T_6_	T_7_
Hen-day egg production (%)							
24-26	72.28^a^±0.91	73.53^ab^±0.54	74.55^bc^±0.44	75.86^c^±0.51	73.18^ab^±0.97	74.22^bc^±0.75	75.19^c^±0.67
26-28	74.09^a^±0.60	75.62^ab^±0.54	76.85^b^±0.65	78.18^c^±0.78	75.29^ab^±0.52	76.41^b^±0.52	77.42^bc^±0.56
28-30	75.40^a^±0.60	76.80^b^±1.19	77.76^bc^±1.19	79.25^c^±1.42	76.18^ab^±0.64	76.99^b^±0.77	78.16^bc^±1.00
30-32	77.16^a^±0.60	78.60^ab^±1.45	80.07^b^±1.89	81.58^c^±1.70	77.91^ab^±0.62	78.94^ab^±0.71	80.30^b^±1.27
32-34	77.91^a^±0.62	78.40^a^±0.71	80.02^b^±1.27	82.33^c^±1.16	78.15^a^±0.66	79.51^b^±0.89	80.82^bc^±1.18
34-36	78.85^a^±0.67	81.82^b^±0.70	83.75^bc^±0.87	85.63^c^±0.77	81.30^b^±0.58	82.42^ab^±0.73	83.55^bc^±0.74
36-38	73.71^a^±0.75	75.63^ab^±0.82	76.27^b^±0.80	79.78^c^±0.60	75.08^ab^±0.82	76.22^b^±0.88	77.52^bc^±0.85
38-40	68.42^a^±0.57	68.92^ab^±0.48	69.85^b^±0.44	70.54^b^±0.47	68.58^a^±0.41	69.08^ab^±0.42	69.42^ab^±0.55
Mean egg mass production (g/day/hen)	74.72^a^±0.66	76.16^b^±0.80	77.39^c^±0.94	79.14^d^±0.92	75.70^ab^±0.65	76.72^bc^±0.70	77.77^c^±0.85
24-26	39.58^a^±0.93	40.94^c^±0.41	39.64^a^±0.53	39.82^ab^±0.84	41.11^c^±0.55	40.36^b^±0.90	40.27^b^±0.67
26-28	39.86^a^±0.69	41.15^c^±0.32	40.15^ab^±0.41	40.41^b^±0.68	41.34^c^±0.89	40.86^bc^±0.80	41.07^c^±0.67
28-30	39.90^a^±0.51	41.17^c^±0.94	40.03^a^±0.93	40.39^b^±1.22	40.96^bc^±0.93	40.62^b^±0.55	40.80^bc^±0.75
30-32	40.30^a^±0.84	41.58^ab^±0.94	40.57^a^±0.94	40.87^a^±0.95	42.11^b^±0.62	41.08^ab^±0.69	41.36^ab^±0.69
32-34	40.33^a^±0.53	41.12^b^±0.53	40.23^a^±1.04	42.53^c^±0.80	41.50^b^±0.73	41.12^b^±0.61	41.43^b^±0.96
34-36	40.07^a^±0.62	42.38^c^±0.66	41.58^b^±0.94	42.07^c^±0.61	42.36^c^±0.75	42.05^c^±0.67	42.21^c^±1.04
36-38	40.05^a^±0.72	42.73^c^±0.53	41.01^ab^±0.91	42.07^b^±0.58	42.69^c^±0.56	41.67^b^±0.67	41.98^b^±0.89
38-40	38.02^a^±0.46	39.51^b^±0.80	37.91^a^±0.84	45.37^c^±0.46	39.57^b^±0.77	38.25^a^±0.75	38.15^a^±0.66
Mean egg weight (g)	39.76^a^±0.66	41.32^c^±0.64	40.14^ab^±0.81	41.69^c^±0.76	41.45^c^±0.72	40.75^b^±0.70	40.90^b^±0.79
24-26	54.77^b^±0.96	55.68^c^±0.41	53.18^ab^±0.48	52.50^a^±0.52	56.18^c^±0.87	54.38^b^±0.53	53.56^ab^±0.64
26-28	53.81^bc^±0.61	54.43^c^±0.44	52.25^ab^±0.33	51.69^a^±0.93	54.91^c^±0.44	53.48^bc^±0.65	53.05^b^±0.65
28-30	52.93^bc^±0.59	53.61^c^±0.68	51.48^a^±0.56	50.97^a^±0.38	53.78^c^±0.59	52.77^bc^±0.65	52.21^b^±0.64
30-32	52.24^bc^±0.83	52.91^c^±0.75	50.68^a^±0.43	50.10^a^±0.70	53.35^c^±0.93	52.04^b^±0.90	51.51^ab^±0.48
32-34	51.77^ab^±0.50	52.45^b^±0.67	50.28^a^±1.13	51.67^ab^±0.74	53.11^c^±0.96	51.72^ab^±0.60	51.27^ab^±0.82
34-36	50.83^ab^±0.53	51.71^bc^±0.72	49.65^a^±1.17	49.13^a^±0.73	52.21^c^±0.86	51.02^b^±0.63	50.53^ab^±0.99
36-38	54.34^ab^±0.63	56.51^c^±0.70	53.78^a^±1.15	53.30^a^±0.62	56.87^c^±0.87	54.68^b^±0.64	54.16^ab^±0.82
38-40	55.57^b^±0.63	57.35^c^±1.01	54.28^a^±1.27	53.80^a^±0.76	57.71^c^±1.07	55.38^b^±0.71	54.96^ab^±0.62
Mean	53.28^b^±0.66	54.33^c^±0.68	51.94^a^±0.81	51.64^a^±0.67	54.76^c^±0.82	53.19^b^±0.66	52.65^ab^±0.70

The mean values in same row with different superscripts differ significantly (p<0.05)

### Egg weight

The egg weight (g) was highest (54.33) in T_2_ followed by T_1_, T_3_ and T_4_ treatment groups (Table-3). This improvement in egg weight at 0.5% level might be due to lower percent hen-day egg production at 0.5% level which, consequently increased the weight of eggs because these two traits are negatively correlated [18]. Comparable results were found by Grashorn *et al.*, 2012 [19] and Youssef *et al*. [16] depicted that egg weight was significantly improved by different dietary treatments as compared to control group (basal diet). In contrary to these findings, supplementation of salts of organic acids had no effect on average egg weight [20-22].

### Egg mass production

The egg mass production was significantly (p<0.05) increased in laying hens fed diets supplemented with different levels (0.5%, 1.0%, and 1.5%) of salts of organic acids as compared to laying hens fed control diet (Table-3). Egg mass production values in treatment groups T_2_, T_4_ and T_5_ were significantly (p<0.05) higher from T_1_, T_3_, T_6_ and T_7_ treatment groups but did not show significant difference among themselves. Similar observations were also made by Soltan [23] and Attia *et al*. [24] who observed that egg mass was significantly improved due to increased egg production in layers. By contrary to these findings, no significant difference in egg mass production among different dietary treatments [25].

### FCR

Results of the study revealed that feed intake per dozen egg production was significantly (p<0.05) higher in control (T_1_) group as compared to other treatment groups (T_2_, T_3_, T_4_, T_5_, T_6_ and T_7_). The mean value of feed intake per dozen egg production was lowest (1.83±0.05 kg) in T_4_ and differed significantly (p<0.05) from T_1_, T_2_, T_5_ and T_6_ groups, but did not differ statistically from T_3_ and T_7_ (Table-2). The results of the study revealed that feed intake per kg egg mass production was significantly (p<0.05) higher in T_3_ (1.0% sodium-butyrate) as compared to other treatment groups (T_1_, T_2_, T_4_, T_5_, T_6_, and T_7_) (Table-2).

The results of the present findings are in consonance with the results of Park *et al*. [26] and Grashorn *et al*. [19] who observed higher feed efficiency on supplementation of salts of organic acids in the ration of layers. In contrast to these findings, FCR did not differ significantly by supplementation of organic acids in the diet of layers [15,25]. It was shown that the efficiency of utilization of feed for egg mass production was significantly improved with the addition of 0.5% level of salts of organic acids in the diet of laying hens. This improvement in FCR might be due to increase percent hen-day egg production and egg mass production. However, it might be due to the recovery of damaged cells of the digestive wall and preservation of microbial balance and improved nutrient utilization of hens belongs to supplemented groups [20].

### Economics of supplementation of salts of organic acids in layers

Average feed costs (Rs.) per dozen egg production and per kg egg mass production during progressive weeks of age are given in Table-4. These results revealed the possibility of increasing economic efficiency by supplementing the diets with salts of organic acids in laying hens. There was net saving of Rs. 1.74, 1.29, 0.88, 1.57, 0.85 and 0.58 in T_2_, T_3,_ T_4_, T_5_, T_6_ and T_7_ groups, respectively, Similarly, there was a reduction in feed cost per kg egg mass production for Rs. 0.52 and 0.99 in groups T_2_ (0.5% sodium-butyrate) and T_5_ (0.5% calcium-propionate), respectively, in comparison to T_1_ (control) group. However, there was an increase in feed cost per kg egg mass by Rs. 3.83, 3.33, 2.24 and 3.60 in T_3_, T_4_, T_6_ and T_7_ treatment groups, respectively, as compared to T_1_ (control) group. By contrast to these results, Soltan [23] and Rahman *et al*. [20] reported that highest economic efficiency was obtained at higher level (720 ppm) of inclusion of organic acids mixture in the diet of layers.

**Table-4:** Average feed cost (Rs.) per dozen egg production and per kg egg mass production during progressive age (weeks) under different dietary treatments.

Parameters (Weeks)	Treatment						

	T_1_	T_2_	T_3_	T_4_	T_5_	T_6_	T_7_
Feed cost (Rs.) per dozen egg production							
24-26	44.23	43.54	44.04	44.56	43.48	44.31	44.65
26-28	44.46	42.41	42.79	43.38	42.58	43.39	43.71
28-30	43.57	41.73	42.09	42.44	41.90	42.46	42.53
30-32	43.12	41.27	41.63	41.97	41.44	42.23	42.30
32-34	42.45	41.05	41.40	41.73	40.99	41.77	42.06
34-36	41.79	40.14	40.70	41.02	40.31	41.08	41.59
36-38	46.23	44.22	44.64	45.03	44.62	45.23	45.59
38-40	47.34	44.90	45.56	45.98	45.30	45.92	46.06
Mean	44.14	42.40	42.85	43.26	42.57	43.29	43.56
Difference Feed cost (Rs.) per kg egg mass production	0	−1.74	−1.29	−0.88	−1.57	−0.85	−0.58
24-26	64.24	63.50	68.92	70.03	63.19	66.93	68.85
26-28	65.13	64.18	68.23	69.32	63.42	66.93	67.91
28-30	65.35	64.86	69.85	68.38	65.68	67.85	69.09
30-32	65.57	65.77	69.39	69.56	64.32	68.31	69.56
32-34	66.91	66.90	71.00	68.61	66.59	69.00	70.03
34-36	68.02	65.54	68.92	69.79	65.68	67.85	69.09
36-38	67.35	65.18	69.15	64.13	63.87	67.16	68.38
38-40	66.02	68.49	73.78	75.45	67.95	72.47	74.49
Mean	66.07	65.55	69.90	69.40	65.08	68.31	69.67
Difference	0	−0.52	3.83	3.33	−0.99	2.24	3.60

The mean values in same row with different superscripts differ significantly (p<0.05)

## Conclusions

From the results of investigation, we can conclude that supplementation of sodium butyrate and calcium propionate was no effect on average feed consumption and body weight gain in layers. Supplementation at 1.5% (T_4_) level of sodium butyrate improved percent hen-day egg production, egg mass production and feed intake per dozen eggs, while 0.5% (T_2_) level improved egg weight and feed intake per kg egg mass production. In overall, supplementation of sodium butyrate and calcium propionate improved persistency of lay, egg weight, and FCR. The addition of salts of organic acids increased the cost per kg feed, but the beneficial effects of salts of organic acids as increased hen-day egg production, improved egg mass production and improved FCR leads to decreased feed cost value per dozen egg and per kg egg mass production at 0.5% level of inclusion. Supplementation of salts of organic acids had no effect on average feed consumption and body weight gain in layers. Supplementation of salts of organic acids in the diet of layers significantly improved production performance (percent hen-day egg production and egg mass production) and FCRs (feed intake per dozen egg and per kg egg mass). Include that this is a preliminary study and positive and negative control will be included in the future study.

## Authors’ Contributions

RD and RSB have planned the study. RD and CSP recorded the information and analyzed the data. SS, L and CSP provided help in the analysis of data. RSB and CSP drafted and revised the manuscript. All authors read and approved the final manuscript.
